# U.S. Pharmacist Opinions Regarding the Rescheduling of Hydrocodone Combination Products: A Pilot Study

**DOI:** 10.3390/pharmacy3030129

**Published:** 2015-08-28

**Authors:** Jordan R. Covvey, Andrea R. Pfalzgraf, Peter P. Cohron

**Affiliations:** 1 Division of Clinical, Social and Administrative Sciences, Mylan School of Pharmacy, Duquesne University, 600 Forbes Ave, Pittsburgh, PA 15282, USA; E-Mail: pfalzgrafa@duq.edu; 2 Law Office of Peter P. Cohron, 350 Tartan Dr, Henderson, KY 42420, USA; E-Mail: petercohron@gmail.com

**Keywords:** opioids, regulation, Drug Enforcement Administration, controlled substance

## Abstract

In October 2014, the Drug Enforcement Administration in the U.S. reclassified hydrocodone combination products (HCPs) from Schedule III to Schedule II, initiating one of the most significant and controversial regulatory changes for opioids in recent national history. The aim of the present study was to determine community pharmacist opinions on the effect of the rescheduling of HCPs on their personal practice. A web-based pilot survey was emailed to a convenience sample through online newsletters of professional pharmacy organizations in Pennsylvania, Kentucky and West Virginia in April/May 2015. A total of 62 surveys were initiated, yielding 56 complete responses. More than 75% of respondents noted increases in their workload as a result of the rescheduling of HCPs. Opinions regarding the intended outcomes of rescheduling were only weakly positive, with only 37.5% of respondents believing it has increased safety and 44.6% of respondents believing it has lessened abuse/diversion. For overall attitudes regarding the rescheduling, respondents were split between positive (26.8%), neutral (26.8%) and negative (46.4%). These initial data suggest that pharmacists have encountered barriers in practice resulting from the rescheduling. Further expanded work is necessary to verify these results from the small sample, and to assess the intended effects of the rescheduling upon the safe and effective use of hydrocodone.

## 1. Introduction

Opioid abuse and associated morbidity and mortality continues to be a significant and widespread problem within the U.S. The Department of Health and Human Services reports that deaths from drug overdoses have increased five-fold since 1980, and in 2010, opioids had been involved in almost 60% of cases [1]. The overall societal cost of prescription opioid harms in 2007 was estimated at $55.7 billion, driven by costs resulting from the workplace (46%), healthcare (45%) and criminal justice (9%) [2]. In 2011, the White House issued a document entitled “Epidemic: responding to America’s prescription drug abuse crisis”, which discusses the need for a coordinated policy response of education, prescription tracking/monitoring, proper medication disposal and enforcement aimed to curb the abuse of opioids [3]. Further to this plan, regulatory efforts to increase safe use of these agents have been prioritized by the Food and Drug Administration (FDA), including the implementation of Risk Evaluation and Mitigation Strategy (REMS) programs for extended-release and long-acting opioids [4] and the developing of a regulatory framework for the use of abuse-deterrent product formulations [5].

In the U.S., strong opioid analgesic agents oxycodone and morphine are classified by the Drug Enforcement Administration (DEA) as Schedule II under the Controlled Substances Act, which specifies that they have a currently accepted medical use but a high potential for abuse and that abuse of the drug may lead to severe psychological or physical dependence [6]. Until recently, hydrocodone had been split between Schedule II and III, with the latter category listing hydrocodone combination products (HCPs) with non-narcotic additives which were thought to have less potential for abuse and lower levels of dependence [6]. Products formulated with hydrocodone in combination with acetaminophen (brand names Vicodin®, Lortab®, Norco®) meet this criteria, and accumulated over 135 million prescriptions in 2012 across the U.S. market [7].

Effective with a final rule on October 6, 2014, the DEA rescheduled HCPs from Schedule III to Schedule II, consolidating all hydrocodone-containing products within this classification [8]. An initial petition to the DEA asking for this change was initiated over 15 years ago, with solicitation of comments from the public and healthcare professionals since this time marked by variable levels of support. The rescheduling carries several important implications for practice, including increased limitations in how physicians can issue prescriptions (e.g., reduction from 180 to 90 day supply with no refills, inability to issue prescriptions by telephone) and requirements for specific inventory requirements, ordering and storage in pharmacies (e.g., “exact counts” of stock supplies, specialized order forms). The implications of this regulation on community pharmacist workload and the downstream effects on patients and medication safety have been a hotly debated topic in the U.S. Therefore, the aim of the present study was to conduct a preliminary survey of community pharmacists in a regional sample to assess their opinions on the impact of the rescheduling of HCPs upon their practice.

## 2. Experimental Section

A short cross-sectional survey was conducted using a questionnaire aiming to assess the effect of the rescheduling of HCPs on community pharmacists. Questions were split into four major sections, including (1) demographic/practice information; (2) policy/practice changes within the pharmacy as a result of the rescheduling; (3) impact upon pharmacy workload, staff and patients; and (4) overall opinions on safety, abuse and the change itself. During survey development, a draft of the survey was distributed to an investigator-selected advisory group of community pharmacists (not involved in the study, or included in later results) to assess for content and face validity based on their practice experience; survey questions were revised based on this feedback to improve the instrument. The survey was then deployed electronically to a convenience sample via email in April and May 2015 using Qualtrics® survey software (Qualtrics; Provo, UT). The survey was included as a section in electronic newsletters distributed to members/supporters of professional pharmacy organizations in the regional geographic area of Pennsylvania, Kentucky and West Virginia. The exact extent of distribution (number of recipients, delivered emails, opened emails) of the three newsletters was not able to be determined through the informal listserv mechanism utilized, therefore calculation of response rate was not possible. The email newsletter included a narrative providing a short introduction to the purpose of the survey and the intended audience (pharmacists working in community settings), with an electronic link to proceed to the survey consent and questionnaire. Two full deployments via the newsletter by each state organization were issued one week apart. Survey responses were collected and analyzed by the investigators using descriptive statistics; Chi-square tests were used to compare differences between subgroups where appropriate. The study was approved by the Duquesne University Institutional Review Board.

## 3. Results

A total of 62 surveys were started and 56 surveys were completed. Respondents were relatively evenly split between male/female and were well represented across the age spectrum (Table 1). Approximately half of respondents were from Pennsylvania, with the remainder broadly split between Kentucky and West Virginia. A significant proportion of respondents were from independent pharmacies in rural settings.

**Table 1:** Survey respondent characteristics.

Characteristic	*n* (%) ^A^
Sex	
Male	30 (53.6)
Female	26 (46.4)
Age group	
Less than 30 years	7 (12.5)
30–39 years	9 (16.1)
40–49 years	11 (19.6)
50–59 years	15 (26.8)
60 year or greater	13 (23.2)
State of practice	
Pennsylvania	27 (48.2)
Kentucky	16 (28.6)
West Virginia	11 (19.6)
Primary practice setting	
Chain pharmacy	17 (30.4)
Independent pharmacy	30 (53.6)
University setting	3 (5.4)
Other	8 (14.3)
Practice location	
Urban	9 (16.1)
Suburban	20 (35.7)
Rural	27 (48.2)

^A^ Percentages reflect denominator of total completed sample (*n* = 56), including non-responses as encountered by the optional nature of the questions.

Regarding the effect of the rescheduling on the daily practice, the majority of respondents noted an increase in time spent dealing with prescriptions and overall workload, as well as perceived concern/difficulty expressed by their patients (Table 2). A total of 44.6% of pharmacists (25 respondents) indicated that they experienced changes in pharmacy policies and practice regarding opioids as a result of the rescheduling. As a result of the rescheduling, 57.1% (32 respondents) have had to contact more prescribers regarding legal issues on HCP prescriptions, 39.3% (22 respondents) have encountered issues regarding the prescribing authority of nurse practitioners/physician assistants and 44.6% (25 respondents) have noted switches for some patients from HCPs to alternative therapies.

**Table 2:** Assessment of changes on pharmacy practice.

Question	Strongly agree/agree, *n* (%) ^A^	Strongly disagree/disagree, *n* (%) ^A^
I spend more time dispensing prescriptions for HCPs	36 (64.3)	8 (14.3)
My support staff (technicians, interns) spend more time dealing with administrative aspects of prescriptions for HCPs	38 (67.9)	9 (16.1)
My overall daily workload in the pharmacy has increased	43 (76.8)	10 (17.9)
My patients/patient caregiver have expressed concern over the change	41 (73.2)	4 (7.1)
My patients/patient caregiver have encountered difficulties obtaining HCPs	38 (67.9)	4 (7.1)
Information regarding legal requirements during the schedule change-over was confusing	22 (39.3)	24 (42.9)

^A^ Percentages reflect denominator of total completed sample *(n* = 56), including “neutral” responses not shown.

While primary practice setting did not influence the assessment of changes in daily practices, the practice location of the pharmacist (urban, suburban or rural) had a consistent effect on response. Those pharmacists in rural settings were more likely to strongly agree/agree across all questions noted in Table 2, while those in urban settings were less likely. For instance, for whether more time had been spent dispensing prescriptions for HCPs, the rate of strongly agree/agree among rural-based pharmacists was 70.4% (19 respondents) compared to suburban pharmacists (12; 60.0%) and urban pharmacists (5; 55.6%). Similarly, for whether overall daily workload had increased, 81.5% (22 respondents) of rural pharmacists strongly agreed/agreed, compared to 75.0% (15 respondents) of suburban pharmacists and 66.7% (6 respondents) of urban pharmacists. Despite these differences, no statistically significant results were noted.

With regards to the effect of rescheduling upon the safety of hydrocodone use, opinions were split among respondents, stating that it will have no effect (31; 55.4%) or will possibly/probably increase safety (21; 37.5%). For abuse/diversion, the opinions were also split between no effect (25; 44.6%) and possibly/probably lessening abuse/diversion (25; 44.6%). For overall attitudes regarding the rescheduling, 15 respondents (26.8%) stated they were positive, 15 respondents (26.8%) stated they were neutral, and 26 respondents (46.4%) stated they were negative. No consistent effect of practice location or setting was noted across these questions.

## 4. Discussion

The rescheduling of HCPs in the U.S. was a contentious regulatory change, and these preliminary data suggest barriers exist among pharmacists post-implementation. Responses indicate generalized negative impact on the majority of respondents’ practices with regards to time and workload, and required changes in practice policies to accommodate the rescheduling. There appears to be weak opinion that the rescheduling may increase safety and lessen abuse/diversion, but overall support for the regulatory change remains low at this early stage within 12 months of the regulation effective date.

In February 2014, the DEA published the proposed rule on the rescheduling of HCPs and solicited opinions from the public during a consultation period; across 573 comments, overall support for the rule was 52%, with the general public (74%) and physicians (56%) higher, and pharmacists/pharmacy students (40%) and ultimate users of HCPs (9%) the lowest [8]. Overall attitudes regarding the rescheduling in this survey were lower, with only about one-quarter of respondents expressing positive opinions. The original basis for the rescheduling was upon scientific and medical evidence supporting the abuse/dependence potential of HCPs as comparable to other Schedule II substances such as oxycodone or morphine. However, dissenting opinions from the DEA public comment docket expressed concern regarding potential for several issues, including detrimental changes in prescribing practices, barriers to patient medication access and economic impacts [8]. Many of these issues were echoed in the present survey, indicating that these concerns were not only perceived prior to the rescheduling, but came to fruition for at least some pharmacists. For instance, the potential for physicians to use alternative therapies instead of prescribing HCPs was noted as a concern in the public comments, for which the DEA responded that “processes and procedures associated with dispensing are not relevant factors to the determination of whether a substance should be controlled or under what schedule a substance should be placed if it is controlled…the DEA believes that when a practitioner makes a medical determination that a particular controlled substance is appropriate to treat a patient’s medical condition, the practitioner will prescribe the appropriate controlled substance, regardless of the substance’s schedule.” [8]. However, results from this survey uncover nearly half of pharmacists seeing some form of switching patients from HCPs to other therapies due to the rescheduling; the clinical effects upon patients are unknown.

A recent survey conducted by the National Fibromyalgia and Chronic Pain Association (NFMCPA) collected data on approximately 3000 patient experiences stemming from the rescheduling and found a host of negative consequences, including “denial of prescriptions to patients, pharmacies refusing to fill prescriptions, higher expenses, extended miles of travel, lost work, and suicidal ideation caused by withdrawal symptoms and untreated pain.” [9]. Fifty two percent of patients reported increased stigma and 30% noted problems filling their hydrocodone prescriptions as a result of the rescheduling [9]. These data are the first reported analyses after the rescheduling and correlate with some of the responses noted in our survey with regards to access to HCPs. However, it also demonstrates that patients with pain have more negative opinions regarding the rescheduling, as was mirrored in the original DEA proposed rule analysis.

The major limitation of the present analysis is the small survey size and sample selection, which diminishes the significance of subgroup analysis. Large survey response among healthcare professionals is a known difficulty due to many factors, including large amounts of requests, limited time for participation and the use of “gatekeeping” for solicitations [10]. Furthermore, convenience sampling was used to solicit responses for this study, which may contribute to response bias. The best method available to reach the community pharmacist audience (through state pharmacy organization newsletters) was unable to allow for an accurate calculation of response rate via email, which limits ability to assess this type of bias. Lastly, this survey measured pharmacist opinions regarding workload, safety, abuse and diversion; objective measures of these metrics would be necessary to confirm the trends suggested in this study, particularly for the trends noted based on the practice location of the pharmacist. With these limitations in mind, this survey was designed as a regional pilot study to assess whether strong opinions on the topic were prevalent among pharmacists, which would warrant continuing inquiry among a wider sample. The authors believe that the results from this project do prompt further analysis, but at present time, should be considered as exploratory and with constraint for external validity.

## 5. Conclusions

This pilot survey of pharmacist opinions regarding the rescheduling of HCPs in the U.S. suggests that a number of barriers have been encountered as a result of the regulation, and that a minority of pharmacists believe the rescheduling has improved safety or lessened abuse/diversion. Soliciting opinions such as these from pharmacy stakeholders should be considered an important aspect of the healthcare policy review process in any country. Expanded work is required to verify these results among a wider population sample. Additionally, there is need to assess the full follow-up impact of the regulation upon the safe and effective use of hydrocodone, including whether levels of abuse/diversion have been positively affected and whether healthcare professionals and patients see long-term disruptions in the treatment of pain.
